# Recent Progress in Upconversion Photodynamic Therapy

**DOI:** 10.3390/nano8050344

**Published:** 2018-05-18

**Authors:** Hailong Qiu, Meiling Tan, Tymish Y. Ohulchanskyy, Jonathan F. Lovell, Guanying Chen

**Affiliations:** 1 College of Functional Crystals, Tianjin University of Technology, 300384 Tianjin, China; qiu@tjut.edu.cn or qiuhl@hit.edu.cn; 2 School of Chemistry and Chemical Engineering, Harbin Institute of Technology, 150001 Harbin, China; tanml@hit.edu.cn; 3 Key Laboratory of Optoelectronic Devices and Systems of Ministry of Education and Guangdong Province, College of Optoelectronic Engineering, Shenzhen University, 518060 Shenzhen, China; tyo@szu.edu.cn; 4 Department of Biomedical Engineering, University at Buffalo, State University of New York, Buffalo, NY 14260, USA; jflovell@buffalo.edu

**Keywords:** photodynamic therapy, upconversion nanoparticles, cancers

## Abstract

Photodynamic therapy (PDT) is a minimally invasive cancer modality that combines a photosensitizer (PS), light, and oxygen. Introduction of new nanotechnologies holds potential to improve PDT performance. Upconversion nanoparticles (UCNPs) offer potentially advantageous benefits for PDT, attributed to their distinct photon upconverting feature. The ability to convert near-infrared (NIR) light into visible or even ultraviolet light via UCNPs allows for the activation of nearby PS agents to produce singlet oxygen, as most PS agents absorb visible and ultraviolet light. The use of a longer NIR wavelength permits light to penetrate deeper into tissue, and thus PDT of a deeper tissue can be effectively achieved with the incorporation of UCNPs. Recent progress in UCNP development has generated the possibility to employ a wide variety of NIR excitation sources in PDT. Use of UCNPs enables concurrent strategies for loading, targeting, and controlling the release of additional drugs. In this review article, recent progress in the development of UCNPs for PDT applications is summarized.

## 1. Introduction

Photodynamic therapy (PDT) is a clinical treatment modality that involves the administration of photo-triggered chemicals known as photosensitizers (PS). PS absorb the light of a specific wavelength and then generate reactive oxygen species (ROS) [1,2,3,4,5,6]. An internalization of PS to cancer cells is beneficial to tumor therapy through photo-triggered killing of tumor cells, as ROS can directly damage cellular organelles such as mitochondria. With the aid of proper laser placement, PDT has been used to treat malignant tumors in different sites of the body, including bladder, prostate, lung, head and neck, and skin cancers. To improve the therapeutic efficacy and reduce the side effects in normal tissue, various PS delivery systems have been established and a variety of luminescent nanomaterials have been employed to guide phototherapy with optical (luminescence) imaging. In particular, semiconductor quantum dots (QDs), metal nanoparticles, and carbon nanomaterials were used for this purpose [7,8,9]. The absorption of PS used in conventional PDT is mostly intense in the blue or ultraviolet (UV) range (i.e., Soret band for porphyrin-related PS), while they are not as absorptive in the deep red range (i.e., 630–695 nm), which is typically used for in vivo applications. In general, PDT is applicable to tumors on or just under the skin or on the lining of internal organs or cavities, but it becomes more complex when treating large and deep-seated tumors. In that case, treatment planning and application of the interstitial approaches in PDT are useful [10].

It is known that tissue has a “window of optical transparency” spanning in the NIR range from ~700 to 1100 nm [11]. NIR light in this region can penetrate significantly deeper into tissues than visible light, because the absorbance and light scattering for most body constituents are lower. Two-photon PDT has been researched for utilizing the simultaneous absorption of two NIR photons to excite a higher lying electronic level corresponding to the visible range [12]. Due to the involvement of a virtual intermediate energy level, the nonlinear two-photon absorption process is relatively inefficient, requiring excitation by an ultra-short pulsed (e.g., femtosecond) laser to provide a high excitation density of ~10^6^ W/cm^2^ in order to generate a useful effect, constraining the application of two-photon PDT in vivo [13]. In contrast, photon upconversion (UC) is known to be a step-wise process that converts NIR light to visible or ultraviolet (UV) emission via the involvement of real intermediate energy levels in lanthanide ions doped into an appropriate host lattice [14,15,16]. The light frequency conversion efficiency in this case is orders of magnitude higher than that of a nonlinear two-photon absorption mechanism [16], and, thus, NIR light upconversion can be achieved with an excitation density of 10^−1^–10^2^ W/cm^2^ provided by an inexpensive low energy continuous-wave (CW) diode laser. This conversion can be readily realized in lanthanide-doped upconversion nanoparticles (UCNPs) which are able to emit shorter wavelength photons under excitation by NIR light. They hold the promise for a new generation of optical probes with great potential in biomedical imaging. In addition, compared with traditional downconversion fluorescent probes, such as quantum dots and organic dyes, UCNPs have prominent advantages such as narrow emission peaks, large stokes shifts, low toxicity, and good photostability, as well as absence of the autofluorescence in the anti-Stokes spectral region, where the upconversion emission is manifested. All these features significantly improve the signal-to-noise ratio in optical bioimaging. In the past few years, a number of groups have been exploring UCNPs as optical nanoprobes in biomedical imaging and detection [13,14,15]. UCNPs-based cancer therapies, particularly NIR-light induced photodynamic therapy, have also been successfully demonstrated in vitro and in vivo. This review aims to summarize the progress achieved to date on the use of luminescent UCNPs for photodynamic therapy in cancer treatment.

## 2. Principles of Photosensitization

Since the discovery of the photodynamic effect in the early 1900s [17,18], great efforts have been devoted towards the development of photosensitizing agents, which exhibit specific photophysical and tissue distribution properties. PDT is a kind of a therapy that takes advantage of the activation of PS by a particular type of light, followed by the subsequent generation of short-lived ROS to kill malignant cells nearby. PDT has been approved as a treatment modality for several types of cancer and skin disorders [19]. The main cytotoxic agent in PDT is known to be an excited state of the molecular oxygen, singlet oxygen (^1^O_2_), a highly active ROS that oxidizes biological substrates [20,21,22]; ^1^O_2_ production can be spectroscopically characterized by its emission peak at 1270 nm [23,24]. Photosensitized oxidation of cell constituents like proteins and DNA induces pathological effects causing cell damage and death. Natural singlet oxygen generation in biological systems is mainly associated with the absorbance of sunlight and dark enzymatic pathways; it can be formed through the photosensitization of aromatic amino acids, such as tryptophan, tyrosine, and phenylalanine, which are abundant light absorbers in the UV range (290–320 nm) [25] or by direct infrared excitation [26]. Singlet oxygen is not only toxic to cells and impairs signaling events, but is also capable of eliciting a cellular stress response. The signaling processes initiated in this response include the activation of mitogen-activated protein kinases [27]. It is worth noting that the closely related non-pathological redox reactions take place during photosynthesis [28].

**Figure 1:**
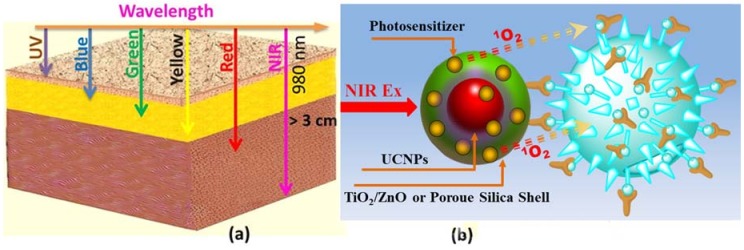
(**a**) Schematic illustration of the penetration depth of different wavelengths in a tissue model; (**b**) Upconverison nanoparticle as a frequency conversion nanotransducer to convert the NIR excitation to visible emission for activation of the photosensitizer, producing reactive singlet molecular oxygen that destroys diseased sites.

In PDT, specificity is achieved first by the enhanced uptake of the PS by a target tissue. Next, the tissue is selectively illuminated, which results in the generation of singlet oxygen by the PS. Thus, PDT is commonly recognized due to its specificity: only cells in close proximity to the photosensitizer are affected, and the PS is not cytotoxic if not illuminated. This specificity could be especially valuable in targeting tumor cells without harming the surrounding tissue. Most PS can be efficiently excited by visible or even UV light, which has a limited penetration depth due to the light absorption and scattering by biological tissues (Figure 1a), resulting in ineffective therapeutic effects for internal or large tumors. UCNPs conjugates may have superiorities, in particular because of the NIR light source and tunable optical properties. The NIR window in the range of 700–1100 nm is known as the window of optical transparency, in which biological tissues have the minimal light absorption, ideal for optical imaging and phototherapy [29,30,31,32,33]. UCNPs have the ability to convert NIR light to visible light, which can then activate PS through the transfer of electronic excitation energy, either radiative (i.e., absorption of UC luminescence photon by PS) or non-radiative (i.e., via Förster or Dexter mechanisms of the electronioc excitation energy transfer). The versatile design of PS for PDT is schematically shown in Figure 1b. Generally, photosensitizing agents that are combined with UCNPs are of two kinds: (1) organic molecules (e.g., porphyrin and porphyrin derivatives); and (2) inorganic materials (e.g., TiO_2_, ZnO). In most cases that use organic molecules as PS, UCNPs are first coated with a polymer or silica shell. Such a shell not only allows for a high payload of the PS, but also protects it from being degraded by the harsh environment. The energy from high-lying excited states of UCNPs will be absorbed by the photosensitizing agents on their surfaces. Subsequently, excited photosensitizing agents will interact with ground-state molecular oxygen in the surroundings, bringing it to the excited singlet state. The generation of reactive singlet oxygen leads to oxidative damage of the cells to which the nanoparticles can be targeted via specific antigen-antibody binding. For the cases of inorganic crystals as PS, they are normally designed as a core-shell architecture comprised of UCNPs as the core and PS (TiO_2_, ZnO) as the shell [34,35]. Hexagonal phase NaYF_4_:Yb^3+^/Er^3+^ or NaYF_4_:Yb^3+^/Tm^3+^ UCNPs are commonly employed as NIR-to-visible nanotransducers, with the crystalline matrix providing the highest photon upconversion efficiency [14]. The Er^3+^ doped UCNPs offer an upconversion emission peak at ~540 and ~660 nm, enabling the activation of organic PS such as Merocyanine 540 (MC 540) [29], zinc phthalocyanine (ZnPc) [36], Chlorine 6 (Ce6) [37], tetraphenylporphyrin (TPP) [38], silicon phthalocyanine dihydroxide (SPCD) [39], Pheophorbide a (Ppa) [40], and Rose Bengal (RB) [41]. On the other hand, Tm^3+^ doped UCNPs emit UV radiation that can trigger the generation of ROS by TiO_2_ and ZnO outer shells [34,35].

## 3. Available Excitation Wavelengths to Excite Lanthanide-Doped UCNPs for PDT Application

Light is a central component in PDT, allowing for PS activation and targeted treatment. To minimize the absorption by endogenous chromophores and reduce the undesired photodamage of a benign tissue, lasers with a narrow bandwidth, which have a well-controlled and focused output, are commonly used for PDT treatment [42,43]. The output laser light can be delivered by optical fibers for the localized application. Different types of lasers have been employed in PDT, including gas, dye, diode, and solid-state lasers, and the wavelengths typically used to excite upconversion emission for PDT applications are in the NIR transmission window (i.e., 980 nm, 915 nm and 808 nm), because absorbance and light scattering for most body constituents are minimal in this range. At present, most of the research work on UCNPs was done using excitation at around 980 nm. Reports of UCNPs for PDT relying on 980 nm light are summarized in Table 1. The imaging penetration depth based on 980 nm excitation has been reported to be as deep as 1.6 cm by Li and co-workers [31], and ~3.2 cm by Chen and co-workers [44]. It should, however, be noted that for imaging applications, the UC emission must be detected outside the imaged object, so the imaging depth is determined by the penetration of both the excitation and emission light. On the other hand, in PDT, only the delivery of the excitation light to the UCNPs matters, as the UC light may be undetectable from outside the tissue while the UC PDT is still efficacious. Correspondingly, the effective treatment depth for UC-based PDT is larger than for UC-based deep tissue imaging.

**Figure 2:**
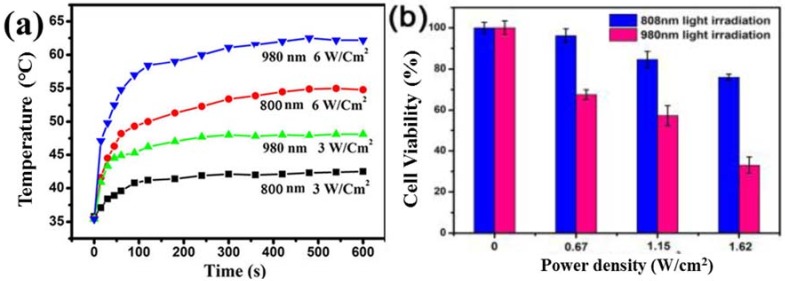
(**a**) Time-resolved temperature in the irradiated nude mouse skins during 10 min irradiation of a 980- and 800-nm laser as a function of different power densities. Reproduced with permission from [58]. Copyright Nature Publishing Group, 2012; (**b**) Heating effects of 808 nm and 980 nm lasers evaluated by the viability of HeLa cancer cells. Reproduced with permission from [59]. Copyright The Royal Society of Chemistry, 2015.

Although 980 nm excitation offers good tissue penetration, water has noticeable absorption at and beyond 980 nm, resulting in tissue heating during PDT treatment with 980 nm light. Development of UCNPs that can be effectively excited by other NIR wavelengths (e.g., 915 nm, as shown by Zhan et al.) may help to solve this problem [55]. Using excitation light in the NIR range but with wavelengths shorter than 980 nm, where water has lower absorption, not only reduces the tissue heating effect, but also allows for deeper tissue penetration in UCNP-based imaging and PDT treatment [55,56,57]. Most recently, 808 nm laser excitation has been introduced with the development of Nd^3+^ sensitized UCNPs with a core/shell structure, which are able to achieve a fairly efficient upconversion of ~800 nm light. Figure 2a illustrates the difference in water heating between 980 and 800 nm laser irradiation. Figure 2b further shows that the difference in water heating causes 808 nm laser light to be less toxic for cultured cells then 980 nm laser light [58,59]. Thus, UCNPs excited by ~800 nm provide a better option for PDT application. Low-cost and high-power laser sources at ~800 nm are widely available. Table 2 summarizes the latest advances in the development of UCNPs-PS systems based on 808 nm excitation for PDT.

**Figure 3:**
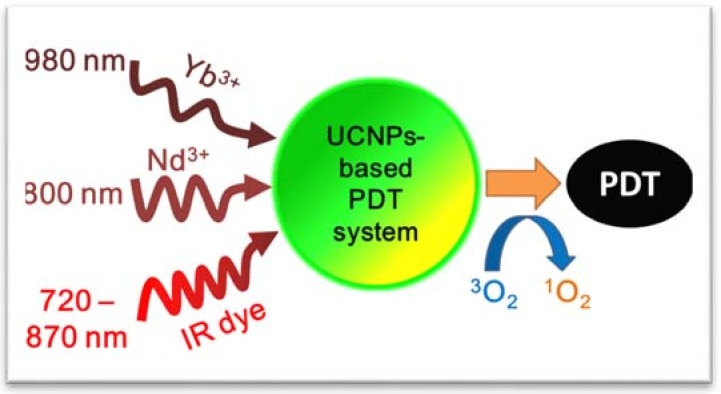
Available and potential excitation wavelengths for UCNPs-based PDT system.

Since lanthanide ions have narrow absorption lines at certain wavelengths, the choice of excitation wavelengths for most UCNPs-based PDT agents is limited; most reported UCNPs-based PDT studies are still confined to conventional Yb^3+^ doped UCNPs using 980 nm and Nd^3+^ doped UCNPs using 808 nm excitation [62,63]. However, this limitation has recently been eliminated by involving organic dyes, broadly absorbing in the NIR range as primary sensitizers. Their broad emission overlaps with the absorption of Yb^3+^ and Nd^3+^, and thus dyes can transfer electronic excitation energy to lanthanides after being excited in their NIR absorption band at ~720–870 nm (Figure 3). Corresponding studies have been reported by our group and other groups [6,64,65]. Our further finding reveals that the infrared (IR) dyes can dramatically boost the upconversion quantum efficiency if a multistep cascade energy transfer is introduced [33]. These results not only allow tissue overheating to be avoided with 980 nm excitation, but also pave the way for the use of variable light sources for upconversion. Indeed, very recently, a complex nanosystem (abbreviated as UCSM) was developed to perform PDT at 808 nm and tracked by upconversion luminescence, computer tomography (CT), and magnetic resonance (MR) imaging, which is composed of mesoporous silica-coated dye-sensitized UCNPs (IR-808-sensitized NaGdF_4_:Yb,Er@NaGdF_4_:Yb @NaNdF_4_:Yb). The visible upconversion emissions at 540 nm and 660 nm simultaneously activate the dual-photosensitizers (MC 540 and Ce 6) to produce a large amount of ROS with a low heating effect, providing an example of an imaging-guided PDT technique [66].

## 4. Emission Wavelengths Offered by Lanthanide-Doped UCNPs for PDT Application

**Figure 4:**
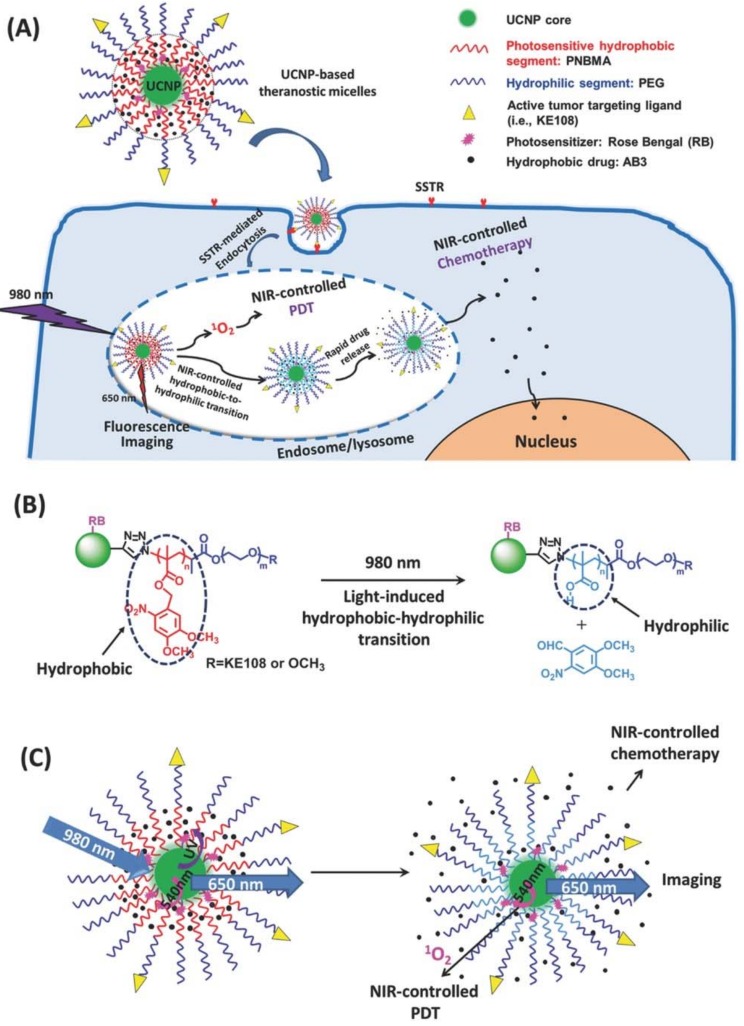
(**A**) A UCNP-based theranostic micelle for simultaneous NIR-controlled combination chemotherapy and PDT, as well as fluorescence imaging; (**B**) An illustration of NIR-triggered hydrophobic-to-hydrophilic transition; (**C**) An illustration of NIR-controlled combination of chemotherapy and PDT, as well as fluorescence imaging. Reproduced with permission from [69]. Copyright Wiley-VCH, 2017.

The wavelengths of emission from lanthanide doped UCNPs, currently employed in PDT applications, are mainly in the visible and UV spectral regions. This is due to the fact that Er^3+^ and Tm^3+^, which are often used as activators in UCNPs, produce emission with the main peaks at 345, 360, 475, 540, and 660 nm [66,67]. These predominant emission bands ensure the interaction with a number of PS agents, as can be seen in Table 1 and Table 2. For example, the first report of a UC PDT application utilized NaYF_4_:Yb,Er UCNP as a core to generate intense visible emission at ~540 nm, which was utilized to donate energy to photosensitizer MC540 incorporated in the outer silica shell [29]. An emission from NaYF_4_:Yb,Er at ~660 nm can also be utilized for PDT, when using zinc (II) phthalocyanine (ZnPc) as a PS, due to a good overlap between the UC emission and ZnPc absorption. Upon excitation by an NIR laser (~980 nm), the UC PDT system is able to generate singlet oxygen to kill murine bladder cancer cells [36]. Additionally, the combination of emissions from both Er^3+^ (540, 650 nm) and Tm^3+^ ions can be simultaneously used, as reported from the nanostructure of NaYF_4_:Yb^3+^, Er^3+^/NaYF_4_:Yb^3+^, Tm^3+^ (450, 475 nm), for PDT applications with C_60_MA as PS that have a broad absorption band [68]. Moreover, different UC emissions can be simultaneously exploited for different purposes. For example, Gong and co-workers developed a multifunctional theranostic UCNP micelle, which emits multiple luminescence bands at 340–370, 540, and 650 nm. The UV peaks overlap with the absorption peak of photocleavable hydrophobic PNBMA segments, triggering a rapid drug release and thus enabling NIR-controlled chemotherapy (Figure 4). RB molecules are activated by luminescence resonance energy transfer at 540 nm to generate ^1^O_2_ for NIR-induced PDT. Meanwhile, the 650 nm emission allows for efficient fluorescence imaging [69]. Note that the hydrophobic core of the UCNP-based theranostic micelle was formed by a photosensitive poly(4,5-dimethoxy-2-nitrobenzyl methacrylate) (PNBMA) polymer that can undergo a hydrophobic-to-hydrophilic transition under NIR-to-UV upconversion by photoinduced cleavage of the polymer side-group. The NIR-triggered hydrophobicto-hydrophilic transition of the micelle core subsequently caused a rapid release of the encapsulated hydrophobic drug, thus leading to a superior anticancer efficacy.

## 5. Surface Modification and Bioconjugation of Lanthanide Doped UCNPs

When it comes to biological applications, particularly PDT, lanthanide doped UCNPs should not only exhibit UC emission, but also have a low toxicity, good monodispersity, and colloidal stability in biological media. Most UC nanocrystals that are synthesized using high-temperature routes with ligands (such as TOPO, oleic acid, and oleylamine) tend to be hydrophobic and lack functional moieties [70,71,72]. Surface modification with hydrophilic ligands is required prior to the chemical attachment of biomolecules. Table 1 lists the representative strategies that are currently being investigated by several different methods to provide UCNPs with dual surface properties (solubility and functionality) for PDT applications.

**Figure 5:**
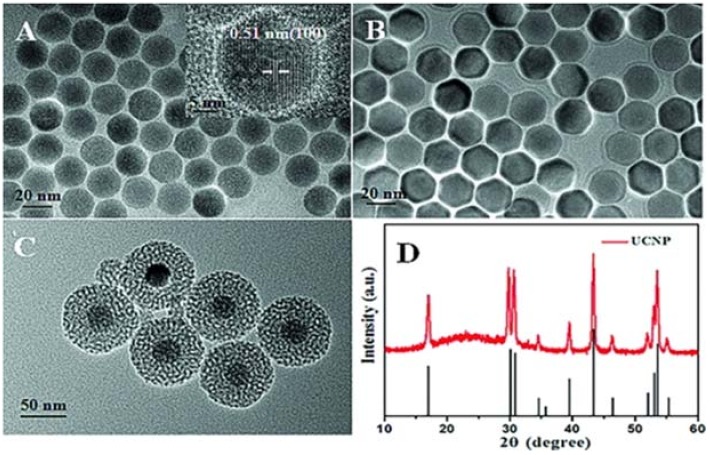

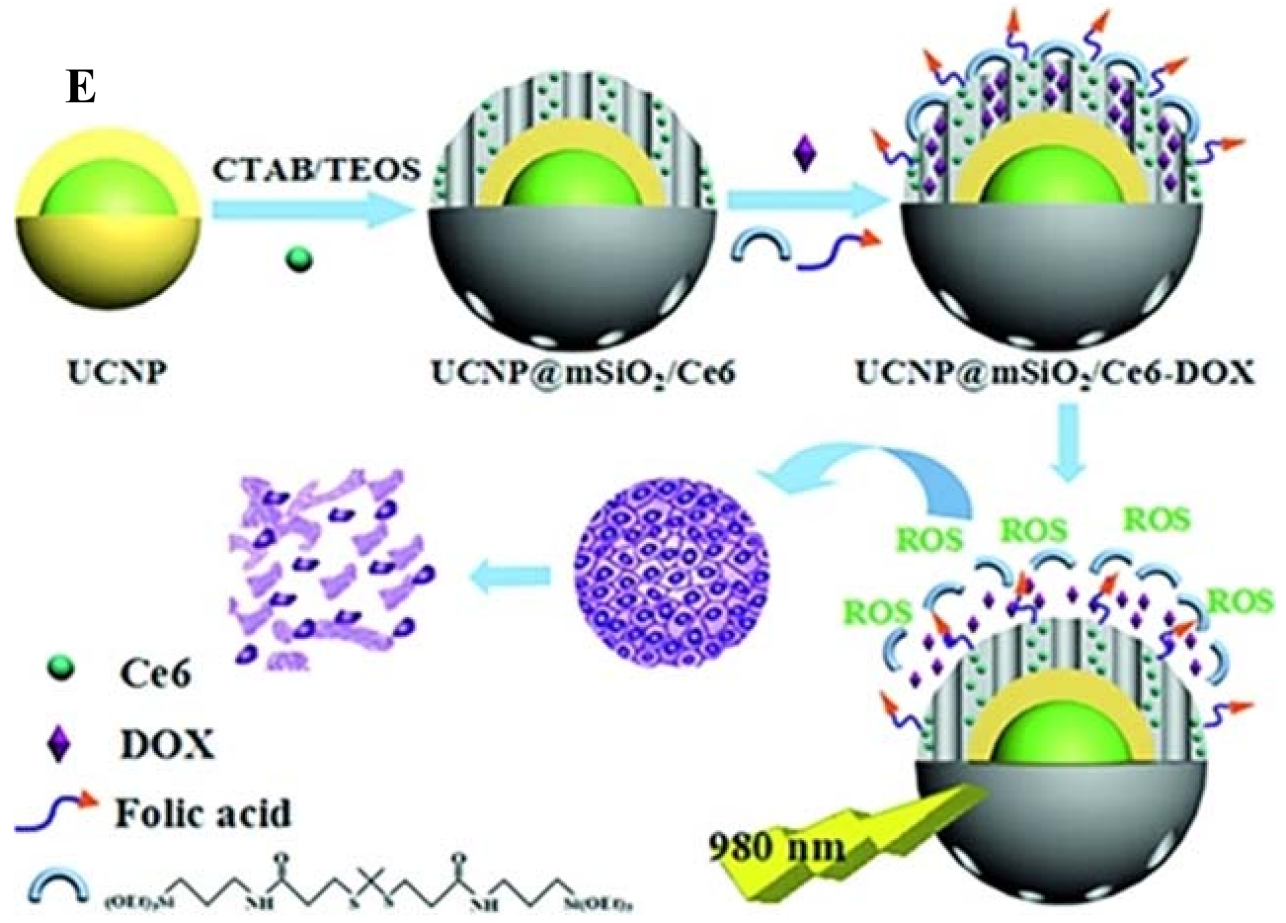
TEM images of (**A**) NaYF_4_:Yb,Er; (**B**) NaYF_4_:Yb,Er@NaYF_4_ (UCNP); (**C**) UCNP@mSiO2/Ce6; (**D**) Powder X-ray diffraction (XRD) pattern for the UCNP and the calculated line pattern for the hexagonal NaYF4 phase. Inset: HRTEM image of NaYF_4_:Yb,Er shows distinct lattice fringes with an interplanar spacing of 0.51 nm ascribed to the (100) plane of hexagonal NaYF_4_. (**E**) Schematic illustration of the synthesis and the controlled release process. Reproduced with permission from [73]. Copyright Wiley-VCH, 2016.

Due to the large pore size and high surface area, mesoporous silica and their composites with UCNPs have attracted considerable attention toward this regard. UCNPs coated with porous silica shells were used as functional delivery carriers and hydrophilic supports [29,47]. In such formulation, incident NIR laser light was upconverted by UCNPs into appropriate light, which excited PS in a convenient way to produce singlet oxygen from dissolved molecular oxygen in the micro-environment. Alternatively, in a recent study, Rose Bengal (RB) PS were covalently bonded to UCNPs. Both the PS loading capacity and the energy transfer efficiency from nanoparticles to PS were significantly improved [41]. Another approach was also reported in the literature: Adsorption of chlorine 6 (Ce6) onto PEGylated UCNPs, forming a UCNP-Ce6 nanocomplex, which was able to enter cancer cells and induce cell death after irradiation with NIR light. This approach was the first to demonstrate highly efficient NIR induced PDT treatment of tumors in vivo, using a mouse model and intratumoral injection of UCNP-Ce6, followed by NIR light exposure [37]. The inorganic PS TiO_2_ has also been applied to PDT, and their growth on UCNPs is mainly carried out in two ways, grafting a number of small TiO_2_ nanoparticles onto the surface of UCNPs or directly coating a TiO_2_ layer onto UCNPs [34,51,52,53,54]. In spite of the good biocompability of TiO_2_, the further surface modification of UCNPs@TiO_2_ core/shell nanoparticles is necessary to hamper the formation of aggregates, facilitating the uptake into cells. In addition, the approach which prepared UCNPs@TiO_2_ core/shell nanoparticles also turned out to be a facile synthetic strategy for the fabrication of UCNP@ZnO core/shell nanoparticles for PDT [35]. Note that the nanoparticle surface can also be exploited to combine different therapeutics, as illustrated in Figure 5, whereby a core–shell nanostructured UCNP@mSiO_2_ with a mesoporous silica shell is used for the storage of two types of guest molecules, i.e., Ce6 for PDT, and doxrorubicin (DOX) for chemotherapy. A thioketal linker was coated on the outside of the core–shell structure by a simple silane coupling reaction to ensure the drug was not prematurely released. Moreover, the targeting ligand of folic acid (FA) can also be immobilized on the surface of the nanoparticles to enhance tumor-selective targeting and internalization. Upon NIR irradiation, the visible light emission (derived from UCNP) can excite Ce6 to generate reactive oxygen species (ROS) to yield irreversible damage to the cancer tissue by the PDT effect, as well as by the chemotherapeutic effect via the release of DOX [73].

## 6. In Vitro and In Vivo PDT by Lanthanide Doped UCNPs

With the rapid progress in developing PDT using lanthanide doped UCNPs, there is a pressing demand for assessment of the potential hazards of these nanoparticles to humans and other biological systems. Yan et al. reported in vitro and in vivo toxicity assessments of water-soluble NaYF_4_ UCNPs and showed that these UCNPs are of a low cytotoxicity [39]. Li et al. reported polyacrylic acid (PAA)-coated UCNPs as luminescence probes for long-term in vivo distribution and toxicity studies. Biodistribution studies revealed that PAA-UCNPs uptake and retention took place primarily in the liver and the spleen and that most of the PAA-UCNPs were excreted from the body of mice in a slow manner. In addition, histological, hematological, and biochemical analyses were used to further quantify the potential toxicity of PAA-UCNPs, and the results indicated that there was no overt toxicity of PAA-UCNPs in mice at long exposure times. The in vitro and in vivo studies all suggest that UCNPs are an excellent NIR emission probe with a low toxicity [74].

**Figure 6:**
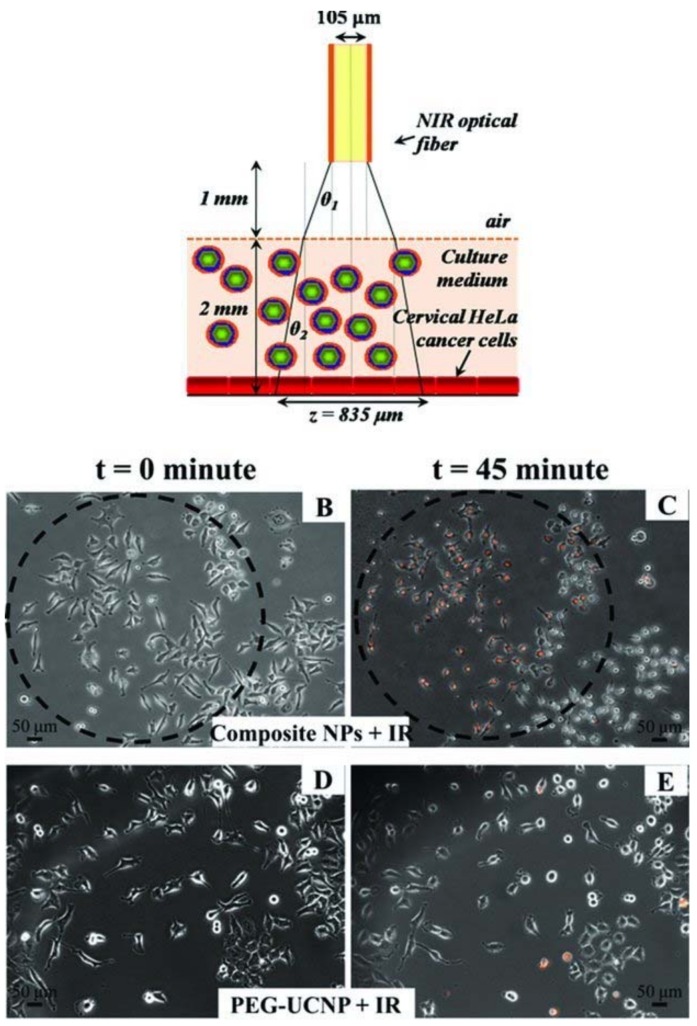
Schematic drawing of the optical fiber placement and illuminated beam sizes in the cell culture experiments (Figure 6A). Cell killing images before and after NIR laser exposure (Figure 6B–D). The black guiding circle represents the region of NIR exposure, which has a diameter of 835 μm as obtained from the calculation above. Figure 6B, C represent the cell killing of the HeLa cancer cells incubated with composite nanoparticles (250 ng L^−1^) containing both UCNP and TPP. At *t* = 0 there is no killing of cells, but after 45 min of 978 nm illumination at 134 W cm^−2^, 75% of the cells in the illuminated region have red fluorescence indication cell death. Figure 6D and E show the control, in which incubation with the PEG-coated UCNP particles without the TPP photosensitizer showed almost no death after 45 min exposure. The images are superimposed fluorescence and phase contrast images of the cells. Reproduced with permission from [38]. Copyright Wiley-VCH, 2011.

To date, the β-NaYF_4_ nanocrystals are known to provide the highest upconversion efficiency among numerous kinds of UCNPs, and these nanocrystals co-doped with Yb and Er are frequently applied in PDT. The NaYF_4_:Yb,Er can emit at 540 nm and 660 nm, which are suitable for absorption by many PS used in PDT, including MC 540, ZnPc, SPCD, and Ce6 (Table 1). PDT using UCNPs was first reported for the combination of silica-coated NaYF_4_:Yb,Er UCNPs and MC 540. In this study, the UCNPs conjugated with anti-MUC1 (episialin) are used for the targeted binding of anti-MUC1 with Episialin (MUC1) present on the surface of the cancer cells. O-carboxymethylated chitosan co-conjugated with pyropheophorbide (Ppa) and RGD peptide c(RGDyK) is used to wrap NaYF_4_:Yb,Er UCNPs in order to improve water solubility, stability, biocompatibility, and resistance to photosensitizers’ self-aggregation during delivery, along with RGD-peptide-based tumor targeting. Ppa is the PS with a maximum absorption at 668 nm, which matches the red emission peak of the NaYF_4_:Yb,Er nanocrystals. The nanoformulation was shown to specifically target tumor cells (U87-MG and MCF-7 cells) and destroy them efficiently under near-infrared laser irradiation [40]. Shan et al. reported the synthesis of composite nanoparticles made by coating UCNPs with *meso*-tetraphenyl porphine (TPP) photosensitizer and the optimized PEG-*b*-PLA block copolymer, and demonstrated the effectiveness of the composite nanoparticles as PDT agents in HeLa cells (Figure 6) [38].

**Figure 7:**
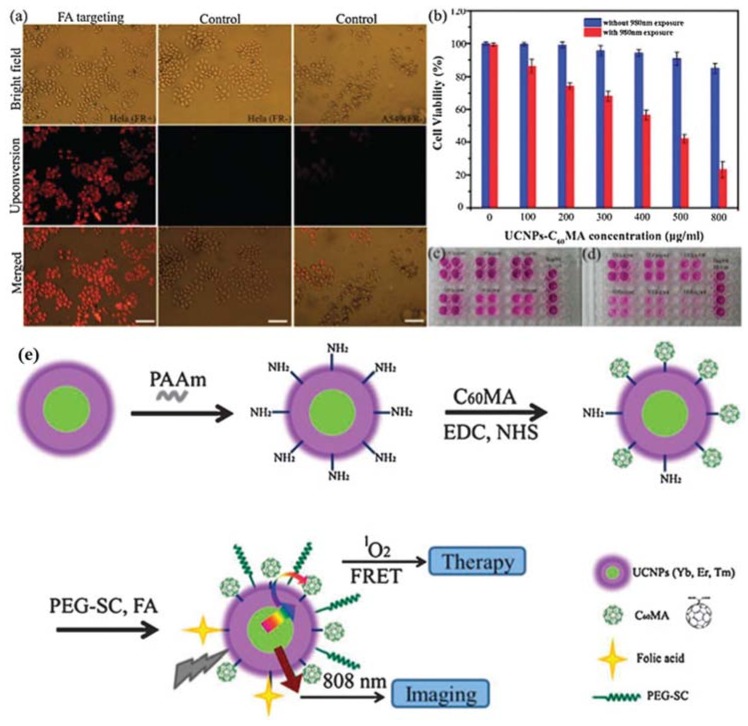
(**a**) Specificity of the UCNPs-C60MA nanoconjugates. Hela cells cultured in folate-free medium (left, positive) and in folate-supplemented medium (middle, negative). The negative control is also performed with A549 cells (right). Scale bar, 50 μm. (**b**) Cell viability of Hela cells 20 treated with UCNPs-C60MA of different concentration with or without 980 nm exposure. (**c**,**d**) The photo of purple formazan dissolved in DMSO, indicating the viability of cells treated with nanoconjugates without 980 nm exposure (**c**) and with 980 nm exposure (**d**). (**e**) The construction and operating principle of the nanoplatform. Reproduced with permission from [68]. Copyright The Royal Society of Chemistry, 2013.

In all of the reported UCNPs-based PDT nanoplatforms, the NaYF_4_:Yb,Er UCNPs have been employed as donors of the electronic excitation energy to excite the acceptor (PS) [32]. The limited spectral overlap between donor and acceptor restricts the ^1^O_2_ production yield. Zhang and co-workers presented a NIR-triggered NIR imaging-guided PDT nanoplatform based on multiplexed FRET, in which multicolor UCNPs are used as donors and monomalonic fullerene (C_60_MA) as the acceptor. Some of the emitting bands (450, 475, 540, and 650 nm) of NaYF_4_:Yb/Er^3+^@NaYF_4_:Yb/Tm UCNPs can contribute to the transfer of excitation energy to C_60_MA due to the broad absorption band of the latter and thus trigger PDT. At the same time, the 808 nm emission can be used for high-contrast NIR luminescence imaging, as illustrated in Figure 7 [68]. In vitro experiments on cancer cells verify the efficient photodynamic effects of the nanoplatform. As the first demonstration of multifunctional UCNPs-fullerene nanoplatform, this result offers a new possibility in exploring a highly stable and efficient nanoplatform suitable for NIR imaging-guided therapy of cancers.

**Figure 8:**
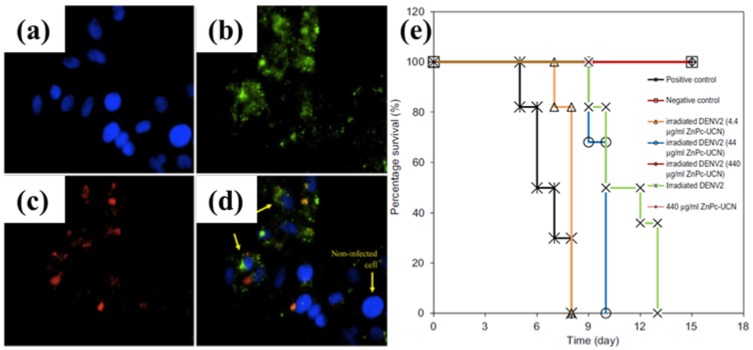
(**a**–**d**) cells were added with anti-DENV2 envelope protein antibody-conjugated ZnPc-UCNPs. Blue fluorescence in (**a**) showed DAPI-stained cell nuclei. Green fluorescence in (**b**) showed FITC-staining of DENV2-infected cells. Red fluorescence in (**c**) showed the location of antibody-conjugated ZnPc-UCNs. (**d**) Kaplan-Meier survival curve of day 1-2 BALB/c suckling mice that were inoculated with photodynamic-inactivated DENV2. The results showed that the UCN-based PDT system can eradicate Dengue virus pathogenesis in BALB/c mice. Reproduced with permission from [50]. Copyright Elsevier, 2012.

The UCNPs effectively reduced the infectious virus titers in vitro with no clear pathogenicity in the murine model and increased the target specificity to virus-infected cells. There is not much research about a promising antiviral approach with feasible applications in the treatments of virus-associated infections, lesions, and cancers. Zhang and co-workers demonstrated the feasibility of UCNP-based PDT to photodynamically inactivate viruses with advantages over current PDT techniques (Figure 8). By carrying the photosensitizer, the ZnPc-UCNPs “solubilize” the highly nonpolar ZnPc [50]. Also, an increased target specificity is achievable as the surface of the nanoparticles can be modified with established protocol for the bioconjugation of targeting moieties such as antibodies or proteins. The strategy demonstrated here further realizes the potential of utilizing these NIR-to-visible UCNPs in the development of promising treatment modality for localized viral infections.

**Figure 9:**
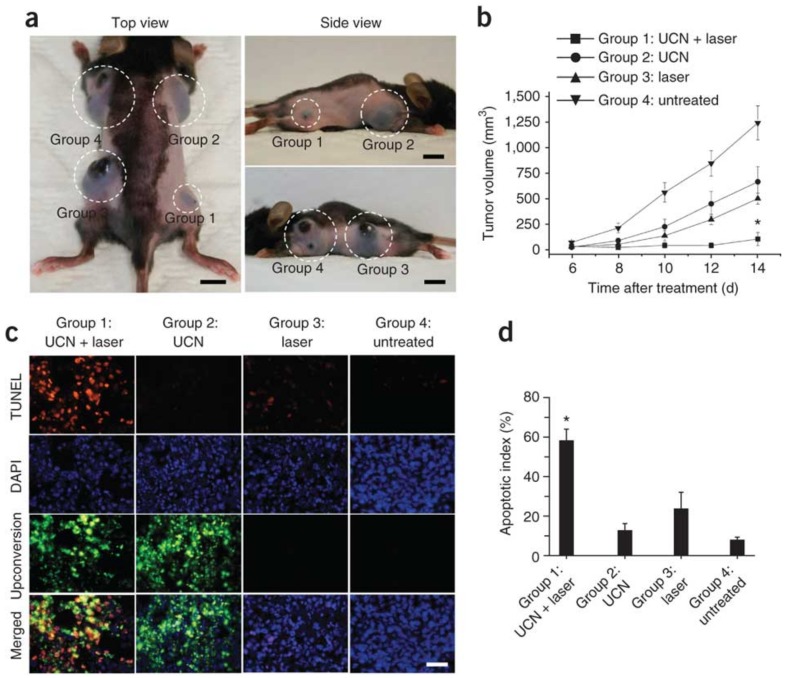
In vivo PDT of injected tumor cells prelabeled with mesoporous-silica–coated UCNPs co-loaded with ZnPc and MC540 photosensitizers. (Excitation by a single wavelength light at 980 nm) (**a**) Representative photos of a mouse showing tumors (highlighted by dashed white circles) at 14 d after treatment with the conditions described for groups 1–4. Scale bars, 10 mm; (**b**) Tumor volumes in the four treatment groups at 6, 8, 10, 12, and 14 d after treatment to determine the effectiveness of the treatment in terms of tumor cell growth inhibition; (**c**) TUNEL staining of tissue sections from the treatment groups at 24 h after treatment to determine the effectiveness of the treatment in terms of tumor cell death by apoptosis. DAPI counterstaining indicates the nuclear region, and upconversion fluorescence imaging indicates the position of the injected UCN-labeled cell (×400 magnification). Scale bar, 20 μm; (**d**) The apoptotic index charted as the percentage of TUNEL-positive apoptotic nuclei divided by the total number of nuclei visualized by counterstaining with DAPI obtained from counts of randomly chosen microscopic fields. Reproduced with permission from [45]. Copyright Nature Publishing Group, 2012.

The first in vivo UCNP-based PDT study in animal experiments was shown by Liu’s group [41]. They loaded Ce6 onto NaFY_4_ with polyethylene glycol (PEG), forming a supramolecular UCNP-Ce6 complex which was used for NIR light-induced PDT treatment of tumors in an animal model. It was found that 70% of tumors were completely eliminated after UCNP-based PDT. Gu et al. reported hydrophilic UCNPs that were prepared by coating the surface of NaYF_4_ UCNPs with N-succinyl-N’-octyl chitosan (SOC) [49]. ZnPc was loaded into the polymer shell via hydrophobic interactions to form a novel drug delivery system for in vivo deep tissue PDT triggered by near-infrared (NIR) light. Zhang and colleagues used a mesoporous-silica-coated fluoride upconversion nanoparticle as a nanotransducer to convert deeper penetrating near-infrared light to visible light which is absorbed by photosensitizers [45]. The layer of the mesoporous silica shell not only allowed a high payload of PS, but also protected the PS from being degraded by the harsh outside environment. Irradiation of the nanotransducers with a 980 nm cw laser resulted in visible upconversion emissions with two main peaks, green (~540 nm) and red (~660 nm), which well matched with the absorption of two photosensitizers, merocyanine 540 (MC540) and zinc (II) phthalocyanine (ZnPc), respectively. A close proximity between the MC540 and ZnPc moieties encased in the mesoporous silica shell and the upconversion core allowed for an efficient energy transfer from the core to the photosensitizers, thereby activating the PS to generate cytotoxic singlet oxygen from the oxygen molecules in the surroundings. A higher PDT efficacy was shown with the dual photosensitizer approach compared to approaches using a single PS, allowing for complete utilization of the frequency conversion process. In vivo studies showed tumor growth inhibition in PDT-treated mice by direct injection of upconversion nanoparticles into melanoma tumors or intravenous injection of upconversion nanoparticles conjugated with a tumor-targeting agent into tumor-bearing mice (Figure 9). Multifunctional nanoparticles have been reported to combine UCL and PDT functions with magnetic properties. A combination of magnetic UCNPs (NaGdF_4_:Yb,Er@NaGdF_4_) with AlC4Pc has recently been used for combined PDT and MR imaging. Here, MEAR cells were incubated with a UCNPs-AlC4Pc complex and irradiated with 980 nm, which resulted in a significant reduction of cell viability, determined by staining with trypan blue, while the magnetic properties of the NaGdF_4_ matrix enabled sensitive MR imaging. A similar result was obtained on the use of NaYF_4_:Yb,Er/NaGdF_4_ core-shell UCNPs for both in vivo luminescence imaging and MRI, while the conjugation with PS delivered a PDT effect [48]. Indeed, it was found that UCNP-photosensitizer was readily accumulated in tumor sites. As such, tumors could be clearly observed not only in the UCL image, but also in the MR image. The shrinkage of tumor size was observed after light exposure at 980 nm. Liu and coworkers reported a novel multi-functional drug delivery system based on UCNPs for targeted drug delivery and cell imaging. The system presented relying on a supramolecular chemistry approach provides a facile and flexible way to load and deliver not only chemotherapeutic molecules such as DOX, but also PS such as Ce6 and TCPP for potential NIR light mediated PDT [75]. A similar result was reported by Zhao’s group [76]. These results clearly indicate that a UCNP-photosensitizer can be used not only as PDT agents for efficient therapy, but also as dual-modal theranostic probes for accurate diagnosis with imaging followed by the therapy.

## 7. Conclusions and Perspectives

UCNPs exhibit great potential for PDT applications. They can be used to sensitize PDT agents through an energy transfer process**.** The mechanism results in the generation of reactive singlet oxygen species that can be used to treat diseased tissues. While UCNPs offer outstanding optical properties and tunable surface chemistries, several issues still remain and need to be addressed before they can be of general practical use in the clinical setting: (i) Current inorganic UCNPs typically have a low upconversion quantum yield of less than 3%, which sets a strong limit for the use of harvested NIR excitation light to produce UV-visible light for the activation of PS. The organic-inorganic hybrid system of dye-sensitized upconversion is emerging to circumvent this problem, which can provide an upconversion quantum yield of up to 9.8% for current under-optimized systems [6,33]; (ii) The Föster resonance energy transfer (FRET) efficiency between the photosensitizer and UCNPs should be further investigated and optimized. The FRET process plays a key role in the determination of the amount of upconverted emissions that can be used for the activation of sensitizers. However, the investigation and optimization of this basic process remains absent; (iii) Most of the current UC PDT systems lack the specific targeting of tumor tissues, due to the lack of targeting ligands on the surface. The introduction of a targeting moiety to the UCNPs-PS nanosystem surface can enhance their retention at tumor sites, thus magnifying the PDT effect; (iv) Lastly, in depth understanding of how UCNPs get transported, metabolized, and excreted, and their compatibility profile in biological environments, is critical before they can be used in vivo as photosensitizers for clinical therapeutic applications.

## Figures and Tables

**Table 1:** A summary of UCNPs-PS PDT systems based on 980 nm excitation.

UCNPs	Coating	Emission (nm)	PS	In Vitro	In Vivo	Ref.
NaYF_4_:Yb/Er	Silica	540	MC 540	MCF-7/AZ breast cancer cells	N/A	[29]
NaYF_4_:Yb/Er	Mesoporous silica	540, 660	MC 540, ZnPc	B16-F0 melanoma cells	B16-F0 cells-bearing C57BL/6 mice.	[45]
NaGdF_4_:Yb/Er@CaF_2_	SiO_2_	660	SPCD	HeLa cells	N/A	[39]
NaYF_4_:Yb/Er	Silica	660	ZnPc	MB49-PSA bladder cancer cells	N/A	[36]
NaYF_4_:Yb/Er	PEG-*b*-PCL	540, 660	TPP	HeLa cells	N/A	[38]
NaYF_4_:Yb/Er	PEG	540	TPP	N/A	N/A	[46]
NaYF_4_:Yb/Er	Mesoporous silica	540	MC 540	murine bladder cancer cells (MB49)	N/A	[47]
NaYF_4_:Yb/Er	PEG	660	Ce6	HeLa cells	tumor-bearing mice	[37]
NaYF_4_:Yb/Er	PEI-OCMC	660	Ppa	integrin positive cells(U87-MG), integrin negative cells (MCF-7)	N/A	[40]
NaYF_4_:Yb/Er@NaGdF_4_	PEG	660	Ce6	U87MG glioblastoma cells	U87MG tumor-bearing nude mice	[48]
NaYF_4_:Yb/Er@NaYF_4_:Yb/Tm	PEG-SC	450, 475, 540, 660	C_60_MA	HeLa cells	N/A	[37]
NaYF_4_:Yb/Er	SOC	660	ZnPc	MCF-7 cancer cells	S180 tumor-bearing Female Kunming mice	[49]
NaYF_4_:Yb/Er	AEP	540	RB	JAR choriocarcinoma cells	N/A	[41]
NaYF_4_:Yb/Er	PEI	660	ZnPc	DENV2-infected HepG2 cells	DENV2 virus-bearing BALB/c mice	[50]
NaYF_4_:Yb/Tm@NaGdF_4_:Yb	PVP	345, 360, 450, 475	TiO_2_	HeLa cells	HeLa tumor-bearing Balbc/c nude mice	[34]
NaYF_4_:Yb/Tm	PEG-1500, APTS	345, 360, 450, 475	TiO_2_	MCF-7 and MCF-7/ADR cells	Female balb/c nude mice	[51]
NaYF_4_:Yb/Tm	Silica and Mal-PEG-silane	345, 360, 450, 475	TiO_2_	OSCC	Female balb/c nude mice	[52]
NaGdF_4_:Yb/Tm	Silica and APTS	345, 360, 450, 475	TiO_2_	HeLa and MCF-7 cells	MCF-7 tumor-bearing nude mice	[53]
NaGdF_4_:Yb/Tm	Silica and hyaluronic acid	345, 360, 450, 475	TiO_2_	MDA-MB-231 cancer cells	N/A	[54]
NaYF_4_:Yb/Tm	Sodium citrate	450, 475	ZnO	MDA-MB-231 breast cancer cell	N/A	[35]

Abbreviations: MC 540: merocyanine 540; ZnPc: zinc (II) phthalocyanine; SPCD: silicon phthalocyanine dihydroxide; PEG-*b*-PCL: poly(ethylene glycol)-*block*-poly(caprolactone); TPP: *meso*-tetraphenyl porphine; PEG: polyethylene glycol; Ce6: Chlorin e6; Ppa: Pyropheophorbide; PEI-OCMC: Polyethylenimine-Ocarboxymethyl chitosan; PEG-SC: polyethylene glycol-succinimidyl carbonate; C_60_MA: monomalonic fullerene; SOC: N-succinyl-N′-octyl chitosan; RB: rose bengal; AEP: 2-aminoethyl dihydrogen phosphate; PEI: polyethyleneimine. PVP: Polyvinyl Pyrrolidone; Mal-PEG-silane: Maleimide-PEG-silane; OSCC cells: human oral squamous cell carcinoma cells; APTS: 3-Aminopropyltriethoxysilane.

**Table 2:** A summary of advances in UCNPs-PS systems based on 808 nm excitation for PDT.

UCNPs	Coating	Emission (nm)	PS	In Vitro	In Vivo	Ref.
NaYF_4_:Yb/Ho@NaYF_4_:Nd@NaYF_4_	PAAm	540	RB	*HeLa cells*	N/A	[59]
NaYbF_4_:Nd@NaGdF_4_:Yb/Er@NaGdF_4_	AEP, PEG	660	Ce6	A549 and KB cells	N/A	[60]
NaGdF_4_:Yb/Tm@NaGdF_4_:Yb@NaNdF_4_:Yb@NaGdF_4_	mSiO_2_	345, 360, 450, 475	TiO_2_	HeLa cells	Female Kunming tumor-bearing mice	[61]
IR-808-dye sensitized NaGdF_4_:Yb,Er@NaGdF_4_:Yb @NaNdF_4_:Yb	mSiO_2_	540, 660	MC540, Ce6	HeLa cells	Female Balb/c	[61]

Abbreviations: PAAm: poly(allylamine); AEP: 2-aminoethyl dihydrogen phosphate (AEP).
